# Are care staff equipped for end-of-life communication? A cross-sectional study in long-term care facilities to identify determinants of self-efficacy

**DOI:** 10.1186/s12904-018-0388-z

**Published:** 2019-01-08

**Authors:** Kirsten Evenblij, Maud ten Koppel, Tinne Smets, Guy A. M. Widdershoven, Bregje D. Onwuteaka-Philipsen, H. Roeline W. Pasman

**Affiliations:** 1 Amsterdam UMC, Department of Public and Occupational Health, VUmc Expertise Center for Palliative Care, Amsterdam Public Health Research Institute, Vrije Universiteit Amsterdam, P.O. Box 7057, 1007 Amsterdam, MB Netherlands; 2 End-of-Life Care Research Group, Vrije Universiteit Brussel (VUB) and Ghent University, Brussels, Belgium; 3 Amsterdam UMC, Department of Medical Humanities, VUmc Expertise Center for Palliative Care, Amsterdam Public Health Research Institute, Vrije Universiteit Amsterdam, Amsterdam, Netherlands

**Keywords:** End-of-life communication, Palliative care, Self-efficacy, Psychiatric nursing, Long-term care facilities, Nurses, Nursing homes

## Abstract

**Background:**

End-of-life conversations are rarely initiated by care staff in long-term care facilities. A possible explanation is care staff’s lack of self-efficacy in such conversations. Research into the determinants of self-efficacy for nurses and care assistants in end-of-life communication is scarce and self-efficacy might differ between care staff of mental health facilities, nursing homes, and care homes. This study aimed to explore differences between care staff in mental health facilities, nursing homes, and care homes with regard to knowledge about palliative care, time pressure, and self-efficacy in end-of-life communication, as well as aiming to identify determinants of high self-efficacy in end-of-life communication.

**Methods:**

Two cross-sectional Dutch studies, one in mental health facilities and one in nursing and care homes (PACE study). Nurses and care assistants were invited to complete a questionnaire in 2015. Multivariable logistic regression analyses were performed to identify determinants of high self-efficacy.

**Results:**

Five hundred forty one nurses and care assistants completed a survey; 137 worked in mental health facilities, 172 in nursing homes, and 232 in care homes. Care staff at mental health facilities were the most knowledgeable about the World Health Organization’s definition of palliative care: 76% answered 4–5 out of 5 items correctly compared to 38% of nursing home staff and 40% of care home staff (*p* < 0.001). Around 60% of care staff in all settings experienced time pressure. Care staff had high self-efficacy regarding end-of-life communication with patients: the overall mean score across all facilities was 5.47 out of 7 (standard deviation 1.25). Determinants of high self-efficacy were working in a mental health facility, age > 36, female, with formal palliative care training, and knowledge of the palliative care definition.

**Conclusion:**

Mental healthcare staff knew more about palliative care and had higher self-efficacy in end-of-life communication compared to nursing and care home staff. Educating care staff about providing palliative care and training them in it might improve end-of-life communication in these facilities.

## Background

In European countries, a substantial proportion of people die in long-term care facilities (hereinafter referred to as “facilities”) [1, 2]. In the Netherlands, for example, approximately 35% of people with chronic conditions die in long-term care settings annually [2]. In this country, two types of facilities specialize in caring for older people: nursing homes and care homes. Nursing homes and care homes differ in the availability of on-site physicians and the complexity of care. Physicians are available on site in nursing homes, whereas they are not on site in care homes. Nursing home residents often require more complex care than care home residents. However, both facilities provide on-site care by nurses and care assistants and both populations have life-limiting chronic illnesses and are in the last phase of life [2–6].

Other long-term care facilities specialize in mental healthcare. In the Netherlands, approximately 27,000 individuals with chronic psychiatric disorders are admitted in the longer term or permanently to mental health facilities [7]. Residents of mental health facilities are relatively young. The treatment focuses on psychiatric problems. However, as psychiatric disorders are associated with significantly higher morbidity and mortality rates, mental health facility staff are also involved in the care for seriously ill and dying residents [8–14]. In the Netherlands, 400–450 people with chronic psychiatric disorders die annually in mental health facilities as a consequence of somatic co-morbidity [7]. Around two fifths of the nurses working in these facilities provide palliative care to psychiatric patients [14]. Hence, while mental health facilities and nursing and care homes differ in patient population and care focus, all residents (may) require long-term complex care until death.

As many people die in facilities, providing appropriate palliative care within facilities has been receiving more attention over recent years [4, 15]. Palliative care relieves the suffering and improves the quality of life of both residents with life-limiting illnesses *and* their relatives [16, 17]. This is achieved by early identification and treatment of pain and other problems of a physical, psychosocial, or spiritual nature, using an interdisciplinary approach. Communicating openly about the end of life with both residents and their relatives is an essential aspect of palliative care [16]. End-of-life communication is defined as *“Communication about issues of a physical, psychological, and existential nature raised by the approaching end of life, with the aim of providing the opportunity for residents to cope with their condition, to assess residents’ care preferences, and to relieve suffering”* [18]. Discussing the course of a disease or care preferences with a patient is generally seen as a task for physicians. However these topics may equally well be discussed between nurses and residents once the resident has been informed by a physician or when the residents themselves bring the issues up [19–22]. Nurses spend more time with residents on a day-to-day basis and are, therefore, in a better position to assess the patient’s condition and initiate or follow up end-of-life conversations. This is especially the case in the facilities without on-site physicians.

Previously, end-of-life communication has been demonstrated to decrease aggressive life-sustaining medical interventions, prevent hospitalizations, increase the use of palliative care [4, 23, 24] and increase relatives’ satisfaction with care [25]. Other research indicates, however, that end-of-life conversations in facilities are rarely initiated by care staff [10, 18, 26–29]. One possible explanation is a lack of self-efficacy in initiating such conversations [30–32]. Bandura defined self-efficacy as *“the perceived capability to perform a certain task.”* The greater the self-efficacy, the more likely the individual is to successfully perform this task [33].

According to Bandura’s theory, successful experiences or seeing other people having successful experiences with end-of-life communication (mastery and vicarious experiences) are associated with self-efficacy. Research into determinants of the self-efficacy for nurses and care assistants in end-of-life communication is scarce, but crucial in order to determine how to improve self-efficacy. There is some evidence that knowledge of palliative care and experience with it, adequate staffing levels, and sufficient available time to spend with residents are prerequisites for end-of-life conversations in facilities [4, 34–36]. Until now, there is no evidence about the differences in self-efficacy of care staff at mental health facilities, nursing homes, and care homes. The aim of this study, therefore, was twofold. Firstly, to describe the knowledge of palliative care, the perceived time pressure, and the self-efficacy of nurses and care assistants in mental health facilities, nursing homes, and care homes and to study the differences between them. Secondly, to analyze whether background characteristics, knowledge about palliative care, and time pressure are determinants of high self-efficacy in end-of-life communication.

## Methods

### Design

This paper combined data from two complementary cross-sectional surveys in the Netherlands. The first was performed in mental health facilities and had the broader aim of providing insights into current palliative care practice in Dutch mental health facilities. Results relating to nurses’ experiences with and barriers to providing palliative care to psychiatric patients have been published elsewhere [14]. The second study concerned the Dutch subset of the Palliative Care for Older People (PACE) project. The PACE study was conducted in nursing and care homes in six European countries including the Netherlands (for the study protocol, see Van den Block et al.^6^). Proportional stratified random sampling, which took account of region, facility type and bed capacity, was used to obtain representative samples for Dutch nursing and care homes. Both studies were conducted in 2015. Nursing and care homes were selected using publicly available lists of nursing and care homes in the Netherlands.

### Participants

For mental health staff, survey invitations were sent to all 598 e-mail addresses registered with the psychiatric nursing division of V&VN (Dutch Nurses’ Association). Members of this nursing association had either completed an educational course in nursing or in social welfare work. Of the 598 e-mail addresses, twenty did not work. Eight nurses stated they had insufficient experience or did not want to fill out the questionnaire. Thirty-three nurses did not currently work in a mental health facility and were excluded. As respondents could fill out the survey anonymously, the researchers were unaware of who did and who did not respond. Two e-mail reminders were therefore sent to all 598 e-mail addresses.

In the PACE study, the managers of the facilities sampled received a letter inviting them to participate in this project. In each participating facility, a contact distributed paper questionnaires amongst the care staff employed in the facility and on duty at the time the researcher visited the facility. A total of 851 nurses and care assistants received invitations to participate. By means of a unique identification code, care staff who did not respond could be identified by the site contact, who sent out a maximum of two reminders to non-responders [6]. In both studies, care staff included care assistants and nurses with varying levels of education. Three levels of education were distinguished: low (care assistant), intermediate (licensed practical nurse/certified nursing assistant), and high (registered nurses at bachelor and master level). In mental health facilities, 13% of care staff had been trained in social care, mostly in addition to nursing training.

### Data collection and measurements

Questionnaires were distributed amongst care staff in mental health facilities, nursing homes, and care homes. The questionnaire included background characteristics, including age, gender, level of education, formal training in palliative care (as part of their degree or as additional education), number of years working in direct resident care, and number of hours per week working in direct patient care.

Knowledge of the key components of the World Health Organization’s (WHO) palliative care definition was used as a proxy for knowledge about palliative care and was measured using five questions that were selected from the MOVE2PC Questionnaire [37]. The questions were measured on the original 3-point Likert scale (disagree, do not disagree/agree, agree with the statement). For analysis, the original scale was first converted into a dichotomous scale (“answered according to the definition” and “not answered according to the definition”), as we were mainly interested in nurses who knew the definition of palliative care. Nurses who answered “do not disagree/agree” were not certain whether or not the statement was correct and this category was therefore merged with the group that answered incorrectly. Next, we calculated the sum of the number of correct answers for each individual (range 0 to 5) and categorized the sum into three categories to make a distinction between those whose knowledge of the definition was poor (0–1 correct answers), average (2–3 correct answers), and good (4–5 correct answers).

Time pressure was measured using 5 items assessing satisfaction with the time available for giving care to patients. This measurement was developed by Ruijters and Stevens. Items were scored on the original 5-point scale (ranging from completely agree to completely disagree) which was dichotomized for analysis into “agree” (agree or completely agree) and “do not agree” (disagree, completely disagree, and do not agree/disagree) [38]. Those who did not agree/disagree were categorized into “do not agree,” as they were not sure that the available time was sufficient.

Self-efficacy in end-of-life communication was measured with the communication subscale from the Self-efficacy in End-of-Life Care survey (S-EOLC) [39], which comprises 8 items that were scored on an 8-point scale ranging from 0 (“cannot do at all”) to 7 (“certainly can do”) [34, 39]. Respondents could also choose the option “not my responsibility” (NMR). For respondents who gave that answer to < 4 items, NMR was recoded into “cannot do at all.” These respondents were probably not confronted with these specific items and do not take action when it occurs, which may indicate that they have little or no experience with these items and are therefore unlikely to feel competent. Respondents who answered NMR to ≥4 items were excluded from further analysis as they were not considered to be relevant for the outcome of this study. Accordingly, 54 nurses/care assistants were excluded: 8 (6.6%) working in mental health facilities, 10 (5.8%) working in nursing homes and 36 (13.9%) working in care homes. A single overall mean score was calculated for each setting. Because the mean S-EOLC scores were highly skewed to the left, the item responses were reported in categories 0–3 (very low S-EOLC), 4–5 (below average S-EOLC), 6–7 (high S-EOLC). For the logistic regression analyses, overall mean S-EOLC scores were dichotomized into scores < 6 = lower (*n* = 247) and ≥ 6 = high (*n* = 219). The cut-off point was based on the median score for all care staff (5.75). Staff in the high self-efficacy group had scored their efficacy as a six or seven on all items.

### Data analysis

The survey data were analyzed in SPSS version 22. Frequencies of all variables were calculated to describe the characteristics of the study sample, knowledge about the definition of palliative care, perceived time pressure, and self-efficacy in end-of-life communication. Chi-squared tests were conducted to test for differences between settings. To control for possible confounding in the association between setting and knowledge and between setting and time pressure, logistic regression analyses were conducted in which all background characteristics were added to the model. As the overall mean S-EOLC score was not normally distributed, a non-parametric test (Kruskal-Wallis) was performed to test for differences in the overall mean S-EOLC score between settings. *P*-values < 0.05 were considered to be statistically significant.

Logistic regression analyses were conducted to identify determinants of high self-efficacy (score ≥ 6) in end-of-life communication. First, univariable logistic regression analyses were performed to assess which variables (background, knowledge, and time) were significantly associated with high self-efficacy. Multivariable logistic regression analysis was then performed to create a model that best describes which factors were associated with high self-efficacy. In each step, the variable with the highest *p*-value was excluded from the model until all variables had *p*-values of < 0.05. Variables entered in the model were setting, age, gender, level of education, formal training in palliative care, number of years working in direct resident care and number of hours per week working in direct care, knowledge of the WHO’s palliative care definition, and perceived time pressure. Odds ratios (OR) and 95% confidence intervals (CI) were calculated. As age and years of experience were largely collinear, we chose to enter age in the multivariable model.

## Results

### Background characteristics

Of the eligible 537 nurses working in mental health facilities, 137 filled out (29%) the questionnaire. Of the 851 nurses/care assistants working in nursing and care homes, 440 participated (52%): 178 worked in nursing homes and 262 in care homes (Table 1). In mental health facilities, a higher proportion of respondents were male compared to nursing and care homes: 25.5% versus 7.9 and 4.2% respectively. Across all settings, the majority (69.1–75.9%) of staff were aged over 35. In mental health facilities, 64% of staff had a higher educational level compared to 5.1% in nursing homes and 5.8% in care homes. However, compared to nursing homes and care homes (63.3 and 54.8%), fewer mental care staff had undertaken formal palliative care training (45.3%). Across all three settings, two thirds of care staff had more than 10 years of working experience in direct patient care. Of care staff working in mental health facilities, 67.2% worked at least 32 h a week compared to 32% of care staff working in facilities for elderly people. Significant inter-group differences were found for gender, level of education, palliative care training, and hours worked per week.

**Table 1:** Background characteristics of care staff at mental health facilities, nursing homes and care homes^a^

	Mental health facility	Nursing home	Care home	*p*-value
	*N* = 137	*N* = 178	*N* = 262	
	%	%	%	
Gender				
Male	25.5	7.9	4.2	< 0.001
Female	74.5	92.1	95.8	
Age				
17–35 years	24.1	26.4	30.9	0.170
36–50 years	32.1	40.5	35.5	
> 50 years	43.8	33.1	33.6	
Level of education^b^				
Low	1.5	44.9	47.7	< 0.001
Intermediate	34.5	50.0	46.5	
High	64.0	5.1	5.8	
Palliative care training^c^				
No	54.7	36.7	45.2	0.006
Yes	45.3	63.3	54.8	
Years working in direct patient care				
≤ 10 years	31.4	33.1	34.0	0.873
> 10 years	68.6	66.9	66.0	
Hours per week working in this facility				
< 32 h	32.8	67.4	67.6	< 0.001
≥ 32 h	67.2	32.6	32.4	

^a^Missing values varied between settings: mental health facilities: 1 missing observation (< 1%), nursing home: 1 missing observation (< 1%), care homes: 1–4 missing observations (< 2%). Χ [2]-test to test inter-group differences

^b^Of the care workers in mental health facilities, 18 were (also) trained in social care (1 in the Intermediate and 17 in the High group)

^c^Palliative care training: part of pre-registration nurse training and/or additional education after pre-registration nurse training

### Knowledge of palliative care

Care staff at mental health facilities were the most knowledgeable about the definition of palliative care: 75.9% answered 4 or 5 items correctly compared to 38.2% of care staff working in nursing homes and 38.8% in care homes (*p* < 0.001) (Table 2). Concerning the individual items, the majority of care staff in all settings were aware that palliative care extends beyond treatment of pain (77.5–87.1%) and includes care for residents’ relatives (84.6–94.8%). Large differences were found between care staff at mental health facilities and at nursing and care homes on the items regarding the initiation of palliative care (81% versus 49.4 and 43.5%) and inclusion of spiritual care (88.8% versus 65.2 and 69.6%). Across all settings, only a minority of staff were aware that palliative care and intensive life prolonging treatment can be combined (29.8–50.0%). Significant inter-group differences were found for all individual items except the first (“The aim of palliative care is treatment of pain only”). When controlling for care staff’s background characteristics, significant differences between the settings remained for “Palliative care starts in the last week of life,” “Palliative care includes spiritual care,” and “Palliative care includes care for the resident’s family.” This shows that mental health facility staff were more knowledgeable about the WHO’s definition of palliative care.

**Table 2:** Knowledge of the definition of palliative care^a^

	Mental health facility	Nursing home	Care home	*p*-value
	N = 137	N = 178	N = 262	
	%	%	%	
Items answered according to the definition				
The aim of palliative care is treatment of pain only (disagree)	87.1	77.5	79.2	0.110
Palliative care starts in the last weeks of life (disagree)	81.0	49.4*	43.5*	< 0.001
Palliative care and intensive life prolonging treatment can be combined (agree)	50.0	29.8	33.5	0.001
Palliative care includes spiritual care (agree)	88.8	65.2*	69.6*	< 0.001
Palliative care includes care for the resident’s family/relatives (agree)	94.8	85.4*	84.6*	0.018
Number of correct answers				
0–1	3.4	13.5	10.4	< 0.001
2–3	20.7	48.3	50.8	
4–5	75.9	38.2	38.8	

^a^Missing values varied between settings: mental health facilities: 21 missing observations (15%), nursing home: 0 missing observations, care homes: 2 missing observations (< 1%). Χ [2]-test to test inter-group differences

^*^Significant difference (*p* < 0.05) compared to mental health facilities (reference) in logistic regression analyses, controlling for gender, age, education level, palliative care training, years working in direct patient care, and hours per week working in this facility

### Time pressure

Across the three settings, a large proportion of care staff experienced time pressure (Table 3). Around half of all care staff agreed with the items “I have sufficient time to discuss problems related to residents with colleagues” and “I reckon I would function better if there was less pressure.” Between 63.2 and 69.6% were of the opinion that they do *not* have sufficient time to provide appropriate care and between 69.0 and 83.2% stated that the time they spend doing administrative tasks is *unreasonable* and care for residents could fall short as a result. For the latter item, a significant inter-group difference was found (*p* = 0.017), but this disappeared after controlling for nurses’ background characteristics.

**Table 3:** Time pressure^a^

	Mental health facility	Nursing home	Care home	p-value
	N = 137	N = 178	N = 262	
	%	%	%	
I have sufficient time to provide appropriate care to residents				
Do not agree	63.2	67.8	69.6	0.451
Agree	36.8	32.2	30.4	
The time I spend doing administrative tasks is reasonable and I am sure that residents do not fall short because of it				
Do not agree	83.2	69.0	72.2	0.017
Agree	16.8	31.0	27.8	
I have sufficient time and possibilities for discussing problems related to residents with colleagues				
Do not agree	51.2	44.3	41.2	0.178
Agree	48.8	55.7	58.8	
I have sufficient time for providing direct care to residents				
Do not agree	59.2	63.2	55.8	0.301
Agree	40.8	36.8	44.2	
I reckon I would function better if there was less pressure				
Do not agree	42.4	46.2	50.6	0.302
Agree	57.6	53.8	49.4	

^a^Missing values varied between settings: mental health facilities: 12 missing observations (8.8%), nursing home: 4–5 missing observations (2.3–2.8%), care homes: 2–3 missing observations (0.9–1.3%). Χ [2]-test to test inter-group differences. No significant differences were found after controlling for staff’s background characteristics

### Confidence in Staff’s ability to engage in end-of-life communication

Overall, care staff felt confident about their ability to engage in end-of-life communication with patients and their families (Table 4). This was reflected by high overall mean S-EOLC scores in all settings: mental health facilities = 5.69 (standard deviation (SD) 1.34), nursing homes = 5.26 (SD 1.24), and care homes = 5.52 (1.19) (*p* = 0.003). Considering the individual items, care staff working in mental health facilities scored significantly higher than care staff in nursing homes and care homes on the following items: “Discussing the likely course of a life-limiting illness with resident or their family”, “Discussing general issues related to dying and death,” and “Having a discussion with the resident about their specific concerns about dying and death.” Care home staff scored significantly higher on the items “Responding to residents asking how long they have got to live” and “Responding to residents asking if there will be much suffering or pain”.

**Table 4:** Self-efficacy regarding end-of-life communication (S-EOLC). S-EOLC scored on a scale from 0 (cannot do at all) to 7 (certainly can do)^a^

The following items relate to end-of-life communication, please answer the following end-of-life by circling the number that best reflects your confidence in your own ability to engage in these activities	Mental health facility	Nursing home	Care home	*p*-value
	N = 137	N = 178	N = 262	
	%	%	%	
1. Discussing the likely course of a life-limiting illness with the resident				
0–3	10.6	26.1	20.6	0.006
4–5	25.7	30.4	24.7	
6–7	63.7	43.5	54.7	
2. Discussing the likely course of a life-limiting illness with the residents’ family				
0–3	11.5	27.3	19.3	0.012
4–5	26.5	26.1	22.0	
6–7	61.9	46.6	58.7	
3. Discussing general issues related to dying and death				
0–3	5.3	9.9	9.0	0.030
4–5	22.1	34.2	22.9	
6–7	72.6	55.9	68.2	
4. Having a conversation with the resident about his/her specific concerns about dying and death				
0–3	3.5	9.9	6.7	0.024
4–5	23.0	29.8	19.3	
6–7	73.5	60.2	74.0	
5. Having a conversation with the family about their specific concerns about the residents dying and death				
0–3	8.0	11.8	4.9	0.098
4–5	23.0	25.5	21.5	
6–7	69.0	62.7	73.5	
6. Providing emotional support to the family upon bereavement				
0–3	6.2	3.7	3.1	0.311
4–5	23.9	27.3	20.2	
6–7	69.9	68.9	76.7	
7. Responding to residents asking how long they have got to live?				
0–3	13.3	16.1	7.2	0.034
4–5	22.1	29.2	30.9	
6–7	64.6	54.7	61.9	
8. Responding to the residents asking if there will there be a lot of suffering or pain				
0–3	16.8	14.3	4.9	0.004
4–5	23.0	29.2	30.5	
6–7	60.2	56.5	64.6	
Overall mean score for each setting (SD)	5.69 (1.34)	5.26 (1.24)	5.52 (1.19)	0.003

^a^Missing values varied between settings. Mental health facilities: 16 missing observations (11.7%), 8 respondents (6.6%) were discarded from analyses (≥4 items ‘not my responsibility’ (NMR)). Nursing homes: 7 missing observations (4.1%) and 10 respondents (5.8%) were discarded from analyses (≥4 items NMR). Care homes: 3 missing observations (1.3%) and 36 respondents (13.7%) were discarded from analyses (≥4 items). Χ [2]-test to test inter-group differences

### Determinants of high self-efficacy in end-of-life communication

Univariable analysis showed that setting, age, formal training in palliative care, numbers of years working in direct care, and knowledge of the definition of palliative care were significant determinants of high self-efficacy in end-of-life communication (S-EOLC score ≥ 6, *p* < 0.05) (Table 5). The number of years working in direct care was not significantly associated with high self-efficacy in the multivariable analysis. Care staff who worked in nursing homes had significantly lower odds ratios (OR = 0.36) for high self-efficacy compared to staff working in mental health facilities. Staff aged 36 or older (36–50 years: OR = 2.96; > 50 years: OR = 4.05), who had completed formal training in palliative care (OR = 2.03) or answered four to five items on the palliative care definition correctly (OR = 2.67) had significantly higher odds ratios for high self-efficacy than those who did not.

**Table 5:** Univariable and multivariable logistic regression model for the prediction of self-efficacy with regard to end-of-life communication amongst care staff (row percentages)^a^

	Self-efficacy score		Univariable		Multivariable (backward selection)	
	< 6 *N* = 271%	≥ 6 *N* = 226%	OR (95% CI)	*p*-value	OR (95% CI)	*p*-value
Setting						
Mental health facility (*n* = 113)	42.5	57.5	reference		reference	
Nursing home (*n* = 161)	66.5	33.5	0.37 (0.23–0.61)	< 0.001	0.36 (0.21–0.63)	< 0.001
Care home (*n* = 223)	52.0	48.0	0.68 (0.43–1.08)	0.099	0.76 (0.45–1.27)	0.294
Age						
17–35 years (*n* = 140)	74.3	25.7	reference		reference	
36–50 years (*n* = 187)	50.8	49.2	2.80 (1.74–4.50)	< 0.001	2.96 (1.80–4.86)	< 0.001
> 50 years (*n* = 170)	42.4	57.6	3.93 (2.42–6.39)	< 0.001	4.05 (2.41–6.78)	< 0.001
Gender						
Male (*n* = 55)	60.0	40.0	reference			
Female (*n* = 439)	54.2	45.8	1.27 (0.72–2.24)	0.417		NS
Level of education						
Low (*n* = 167)	55.1	44.9	reference			
Intermediate (*n* = 234)	57.7	42.3	0.90 (0.60–1.34)	0.604		NS
High (*n* = 92)	45.7	54.3	1.46 (0.88–2.44)	0.147		NS
Formal training in palliative care						
No (*n* = 209)	61.7	38.3	reference		reference	
Yes (*n* = 284)	50.0	50.0	1.61 (1.12–2.32)	0.010	2.03 (1.36–3.03)	0.001
Number of years working in direct care						
≤ 10 years (n = 161)	68.3	31.7	reference			
> 10 years (*n* = 336)	47.9	52.1	2.34 (1.56–3.48)	< 0.001		NS
Hours a week working						
< 32 h (*n* = 297)	56.9	43.1	reference			
≥ 32 h (*n* = 200)	51.0	49.0	1.27 (0.89–1.82)	0.195		NS
Knowledge of the palliative care definition						
0–1 (*n* = 48)	75.0	25.0	reference		reference	
2–3 (*n* = 208)	57.7	42.3	2.20 (1.08–4.47)	0.029	2.11 (0.99–4.53)	0.054
4–5 (*n* = 236)	47.5	52.5	3.32 (1.65–6.70)	0.001	2.67 (1.24–5.73)	0.012
I have sufficient time to provide appropriate care to residents						
Do not agree (*n* = 329)	55.0	45.0	reference			
Agree (*n* = 162)	53.7	46.3	1.05 (0.72–1.54)	0.784		NS
The time I spend doing administrative tasks is reasonable and I am sure that residents do not fall short because of it.						
Do not agree (*n* = 366)	53.7	46.2	reference			
Agree (*n* = 125)	56.8	43.2	0.89 (0.59–1.34)	0.564		NS
I have sufficient time and possibilities for discussing problems related to residents with colleagues.						
Do not agree (n = 219)	52.5	47.5	reference			
Agree (*n* = 272)	56.3	43.8	0.86 (0.60–1.23)	0.408		NS
I have sufficient time for providing direct care to residents.						
Do not agree (*n* = 290)	53.1	46.9	reference			
Agree (*n* = 201)	56.7	43.3	0.86 (0.60–1.24)	0.429		NS
I reckon I would function better if there was less pressure.						
Do not agree (*n* = 230)	53.0	47.0	reference			
Agree (*n* = 260)	55.8	44.2	0.90 (0.63–1.28)	0.545		NS

^a^Total *N* = 497: 26 respondents (4.5%) did not fill in the S-EOLC questions, and 54 respondents (9.4%) were excluded from analysis since they answered ‘not my responsibility’ ≥4 times. Missing observations varied per independent variable, ranging from 9 to 17 (1.8–3.4%). NS: not significant: all variables were included in multivariable analyses. Using a stepwise backward selection method, all non-significant variables (*p*-value > 0.05) were excluded. Because of collinearity between age and years of experience, we chose to enter only age in the multivariable model

## Discussion

### Knowledge of palliative care

Overall, care staff were reasonably well-acquainted with the components of palliative care. Staff at mental health facilities were more knowledgeable compared to staff at nursing and care homes, even though a lower percentage of them had completed palliative care training. Some components of palliative care were better known than others. Almost all care staff were familiar with palliative care not being restricted to pain alleviation, but only a minority knew that palliative care and life-prolonging treatment can be combined. One possible explanation is that nurses in these facilities do not have direct experience with intensive life-prolonging treatment. This is especially true for staff in nursing and care homes where the illnesses of most patients, i.e. frail older people and people with dementia, is characterized by a prolonged and gradual decline [40] and where care is primarily focused on the quality of life.

### Time pressure

Across the three settings, around 60% of care staff reported that the available time is insufficient to provide appropriate care. Moreover, the amount of time care staff spent on administrative work was perceived to harm the care for patients. This was especially true for mental healthcare staff. Previous studies have reported high time pressure caused by inadequate staffing levels and the intense and time-consuming nature of palliative care as hindering palliative care in facilities [14, 34, 36]. The shift towards outpatient mental healthcare accompanied by large budget cuts is increasing time pressure in mental health facilities even more [14]. In addition, as a result of changes in the health system, older people suffering from chronic diseases are only admitted to a facility when their care needs are very complex. This again increases the workload and thus the time pressure. As time pressure was high across all settings, this should be tackled in order to ensure appropriate palliative care.

### Factors associated with self-efficacy in end-of-life communication

Across all settings, the care staff reported high self-efficacy in end-of-life communication. Although this may very well be true, it is also possible that some staff may have overestimated their ability to converse about these difficult issues. Due to a lack of self-awareness, people with lower ability to communicate about the end of life may be unable to objectively evaluate their actual competence or lack thereof. This is referred to as the Dunning-Kruger effect [41]. Determinants of high self-efficacy were setting, age > 36, female, formal palliative care training, and knowledge of palliative care. Care staff working in mental health facilities had significantly higher self-efficacy in end-of-life communication compared to care staff working in facilities for older people. This may be due to the experience of mental health staff with psychosocial interventions [42, 43]. Care staff in mental healthcare are also often trained in and accustomed to discussing complex and sensitive topics such as a wish to die [14].

While nursing home care staff are most likely to be confronted with the care for dying residents due to the frail population, their self-efficacy in end-of-life communication was lowest. Care home staff may possibly exhibit higher self-efficacy than nursing home staff because they have to take responsibility for discussing end-of-life issues with residents themselves in the absence of an on-site physician. In nursing homes, staff may be more inclined to leave this responsibility to the on-site physicians. Uncertainty about who should take the responsibility to start end-of-life conversations can hamper its implementation [31, 32].

Not surprisingly, staff trained in palliative care were more likely to have high self-efficacy for discussing end-of-life issues with residents and their families. Earlier studies reported that palliative care training can improve care staff’s confidence with regard to talking about death and dying [32, 44, 45]. Unfortunately, palliative care training has not yet been widely implemented in nursing education programs and in this study only half of the care staff had completed palliative care training. Nursing education programs often focus on acute care nursing and fail to address end-of-life care [4, 46]. As a result, care staff lack knowledge of palliative care and might feel unprepared to cope with dying patients [4, 35].

We also found that staff aged ≥36 had high self-efficacy more often. This is most likely due to the fact that they have more experience with providing palliative care and talking about end-of-life issues. Existing literature states that younger nurses are more afraid of death, have more negative attitudes towards end-of-life care and are less skilled in dealing with emotional work [47, 48]. Palliative care education therefore should ideally incorporate experiential learning in which nurses learn by reflecting on concrete experiences with end-of-life discussions with patients [49].

## Strengths and limitations

To our knowledge, this is the first study that quantitatively assessed care staff’s knowledge of palliative care, the perceived time pressure, and their self-efficacy in end-of-life communication. Also, for the first time, a comparison was made between the staff in mental health facilities, nursing homes, and care homes. Moreover, the study identified determinants for high perceived efficacy in end-of-life communication. This study is, therefore, unique in offering insights into how well care staff in long-term care facilities are equipped to discuss end-of-life issues with residents. There are, however, several limitations that should be acknowledged. Firstly, two databases from independent studies were merged for the analysis. The differences between the three settings might therefore be at least partially induced by the different recruitment method used in the study in the mental health facilities and the study in the nursing and care homes. However, there were many similarities in the methodology: similar participants were recruited (nurses and care assistants), the questionnaires contained the same questions and the data were collected in the same period and country. Secondly, there is a risk of sampling and selection bias, especially for the mental health staff. To recruit mental health staff, a union membership list of the Dutch nurses’ association was used instead of a random sample of mental health staff in the Netherlands. Thirdly, the response rate was fairly low and study participants are more likely to be interested in the topic studied compared to non-responders. Our results might therefore overestimate the actual knowledge and self-efficacy of Dutch facility staff. Lastly, we recruited mental health nurses who provided long-term care to patients. This type of care is mostly provided in mental health facilities, but we cannot rule out the possibility that some nurses provided palliative care in an outpatient setting. It is unknown whether or not this impacted our results.

## Conclusion

This study provides a good starting point for improving end-of-life conversations in long-term care facilities by providing evidence about factors that could improve care staff’s self-efficacy. As higher self-efficacy is associated with an increased likelihood of performing a certain procedure, nursing education programs should pay more attention to palliative care and end-of-life conversations in their curriculum and use more experiential/bedside learning methods to promote successful experience with this in an early stage of working life. Moreover, clear agreements about who should take responsibility for initiating end-of-life conversations should be made, as the absence of agreements might hamper end-of-life conversations.

## Abbreviations

CI: Confidence interval
NMR: Not my responsibility
OR: Odds ratio
PACE: Palliative Care for Older People
SD: Standard deviation
S-EOLC: Self-efficacy in End-of-Life Care
V&VN: Dutch Nurses’ Association
WHO: World Health Organization
